# Is arterial spin labelling necessarily low perfusion for cavernous sinus venous malformation? A case of hyperperfusion cavernous sinus venous malformation

**DOI:** 10.1093/bjrcr/uaad007

**Published:** 2023-12-13

**Authors:** Dan Luo, Xinlan Xiao

**Affiliations:** Department of Radiology, The Second Affiliated Hospital of Nanchang University, Nanchang, Jiangxi 330006, China; Department of Radiology, The Second Affiliated Hospital of Nanchang University, Nanchang, Jiangxi 330006, China

**Keywords:** cavernous sinus venous malformation, meningiomas, ASL

## Abstract

Generally, due to the complexity of the skull base structures, it is difficult to differentiate cavernous vascular malformation and meningioma in the cavernous sinus area using conventional imaging studies. Cavernous sinus venous malformation are characterized by increased capillary masses without a direct arterial supply, typically leading to low perfusion. On the other hand, meningiomas receive arterial blood supply to the tumour and often exhibit high perfusion. So, arterial spin labelling (ASL) can be helpful in distinguishing between the 2 tumour types. However, in our specific case of a cavernous sinus venous malformation, the ASL imaging showed hyperperfusion. Further analysis revealed that this hyperperfusion on ASL can occur when cavernous sinus venous malformation is associated with arteriovenous fistula malformation.

## Background

Extracerebral cavernous vascular malformations mostly occur in the middle cranial fossa.^1^ Currently, MRI scan with contrast is a commonly used imaging modality for diagnosing cavernous sinus venous malformation.^2^ However, the anatomical complexity of the sellar region makes it is difficult to differentiate between cavernous sinus venous malformation and meningiomas based on their appearance on MRI scan with contrast.

There have been reports of a misdiagnosis rate ranging from 66.7% to 87.5% for cavernous sinus venous malformation mistaken as meningiomas.^3^ The utilization of arterial spin labelling (ASL) imaging can aid in differentiating cavernous sinus venous malformation from meningiomas by measuring cerebral blood flow (CBF), particularly in cases where conventional MRI sequences fail to provide a clear distinction between the 2 types of tumours. Typically, cavernous sinus venous malformation consists of increased capillary clusters without a marked arterial blood supply, resulting in low perfusion on ASL imaging. Only one case of extracerebral cavernous malformation^4^ showed obvious high perfusion in ASL when coexisting with arteriovenous fistula. The ASL of this patient showed high perfusion, which is different from the common perfusion of cavernous sinus venous malformation. The objective is to provide valuable insights into the atypical perfusion characteristics of high-perfusion cavernous sinus venous malformation.

## Case

**Figure 1.**
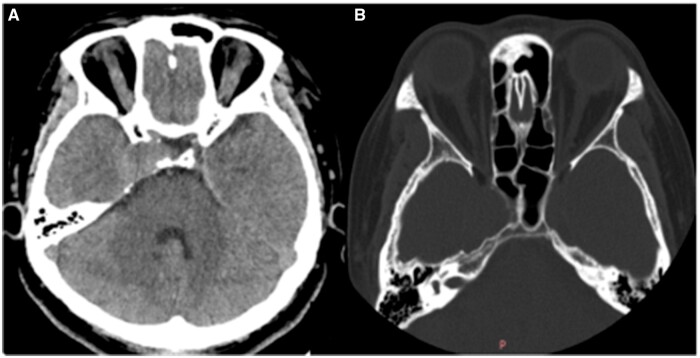
Non-contrast head CT. (A) Soft tissue window showing soft tissue density in the cavernous sinus area. (B) Bone window showing bone remodelling and thinning.

**Figure 2.**
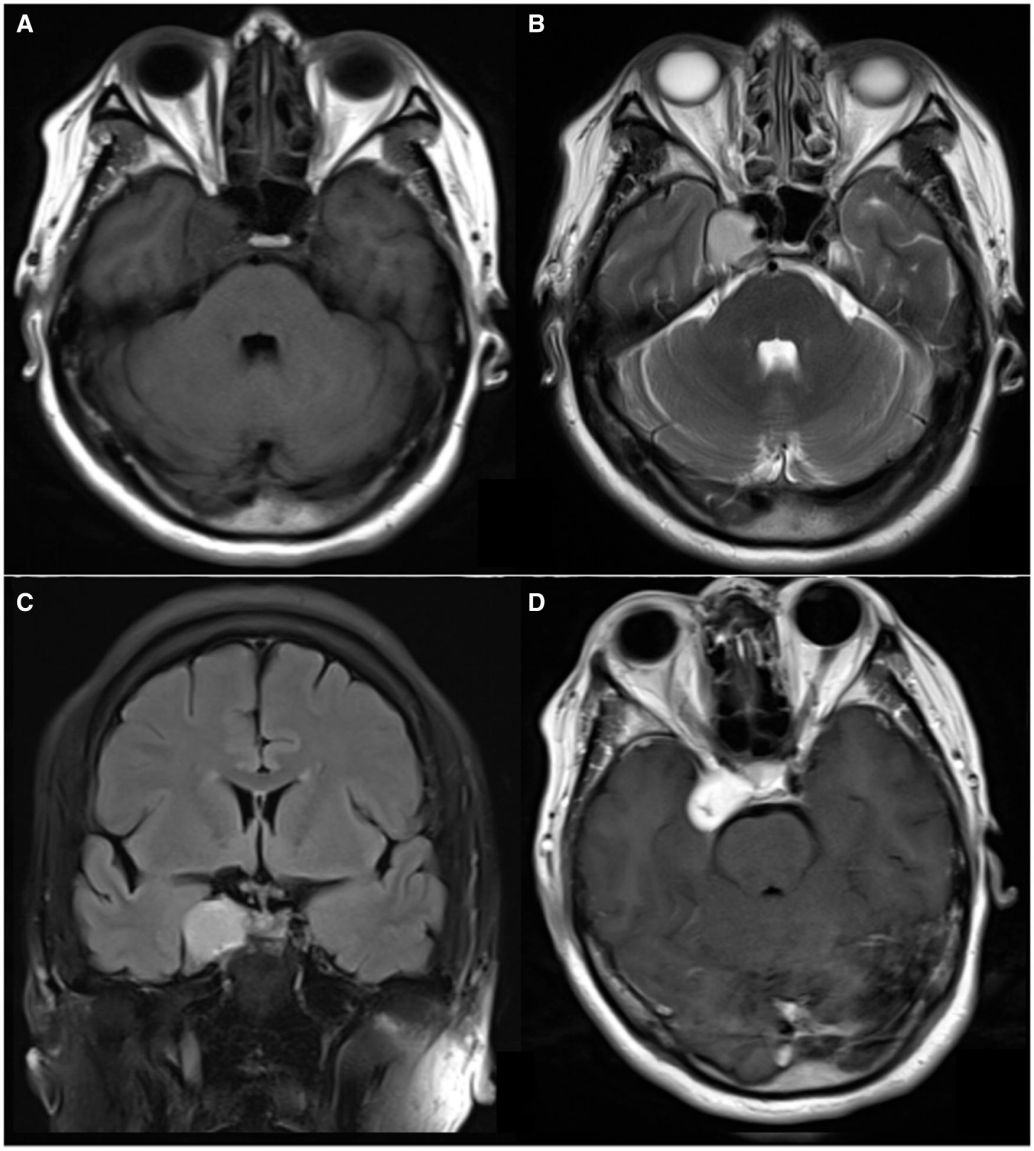
Pre- and postcontrast brain MRI. (A) Hypointensity mass in the cavernous sinus area on TIWI. (B) Hyperintensity mass with uniform internal signal in the cavernous sinus area on T2WI. (C) Hyperintensity mass in the cavernous sinus area on T2 FLAIR. (D) The enhanced scan shows marked and even enhancement of the mass.

**Figure 3.**
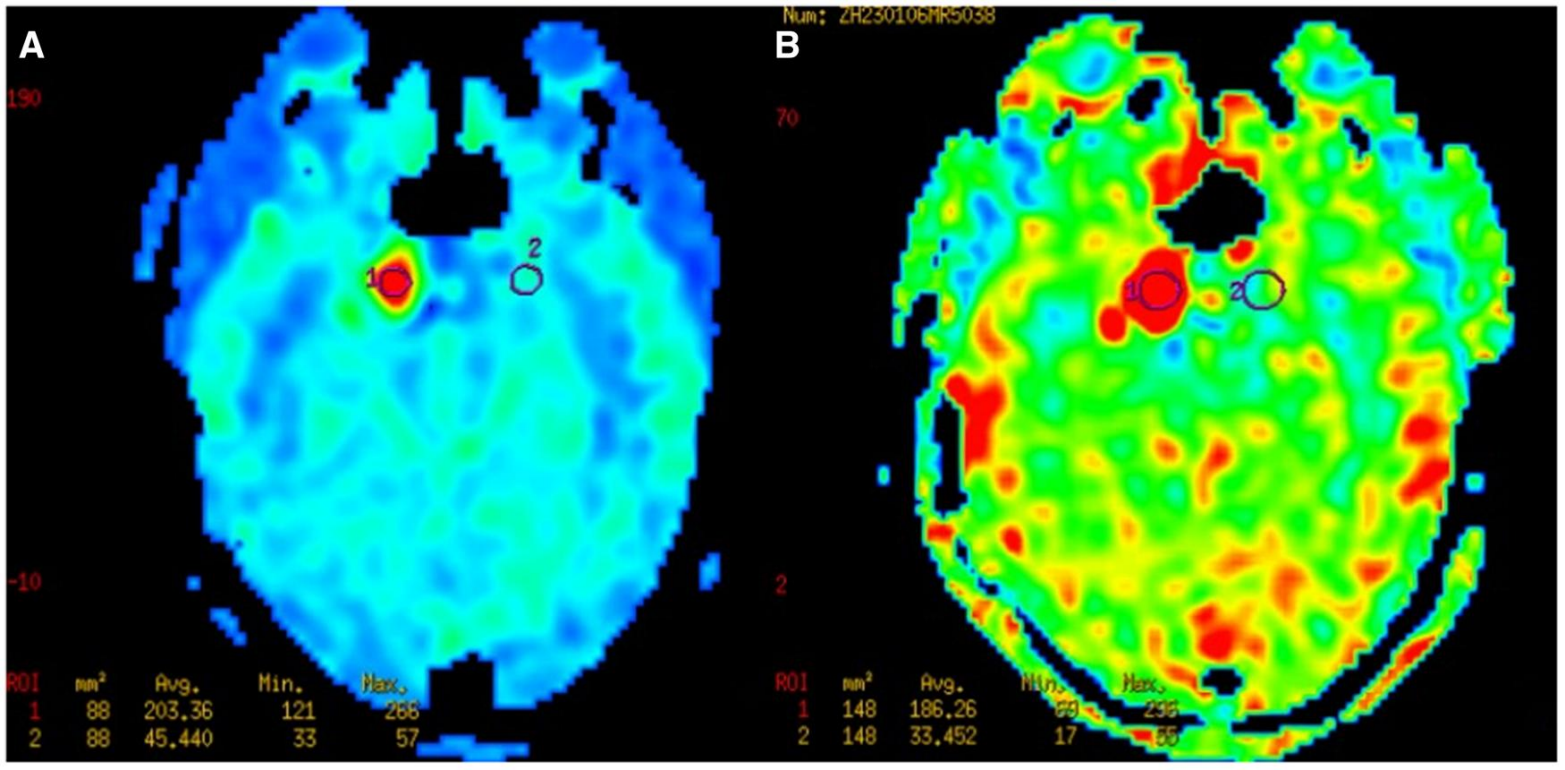
Post-processed CBF map obtained from ASL. (A and B) A post-labeling delay（PLD） of 1.5 s (A) and a PLD of 2.5 s (B), and the measured CBF values were 203.36 (mL/100 g/min) and 186.26 (mL/100 g/min).

A 48-year-old woman presented with a complaint of blurred vision in her right eye persisting for over a month. During the physical examination, restricted external rotation of the right eye and diplopia were observed while focusing on objects. Her CT brain (Figure 1) which revealed a mass adjacent to the right temporal pole, within the cavernous sinus, displaying soft tissue density with a CT value of 42 Hounsfield units (Hu). The mass was found to be connected to the base of the cavernous sinus and exhibited growth towards the skull, resulting in remodelling of the surrounding bone. However, there is no obvious hypertrophic sclerosis in the surrounding skull. MRI imaging (Figure 2) exhibited a lesion characterized by slightly hypointensity on T1-weighted imaging (T1WI) and hyperintensity on T2-weighted imaging (T2WI), which appeared uniformly distributed. The diffusion weighted imaging (DWI) signal was slightly low, while the T2 FLAIR sequence demonstrated marked hyperintensity. Post-contrast MRI displayed prominent and relatively homogeneous enhancement in the region of the right cavernous sinus. The enhancement gradually filled inward. The GE AW4.6 workstation’s Functool software was used for image analysis and data processing, and the CBF map was automatically generated by ASL. The ASL image clearly demonstrates that the perfusion of the mass is considerably higher compared to the perfusion observed in the surrounding and contralateral cerebral cortex (Figure 3).

**Figure 4.**
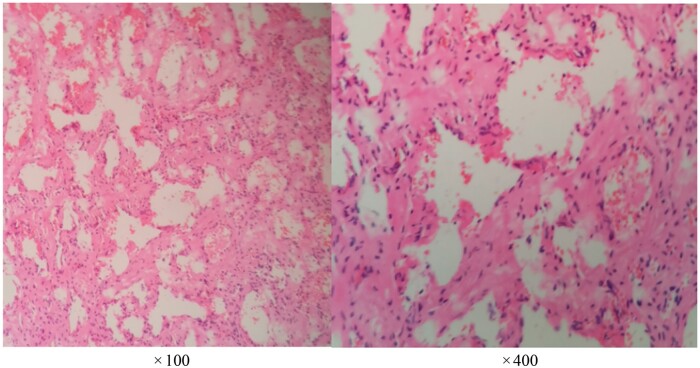
Hematoxylin-eosinstaining （HE）-stained histological section reveals an increased number of blood vessels with varying thickness.

Taking into account the patient’s clinical symptoms and the findings from the imaging examinations, the final imaging diagnosis suggests the possibility of a meningioma. The patient underwent cavernous sinus brain tumour resection under general anaesthesia. Intraoperatively, the tumour was identified in the cavernous sinus area, appearing as a semi-transparent grey-red mass with a diameter of approximately 2 cm. The postoperative pathological report revealed that the lumen was composed of numerous blood vessels, varying in size, with uneven thickness of the vessel walls (Figure 4). The final pathological diagnosis is a cavernous sinus venous malformation.

## Discussion

With the continuous maturity and development of ASL sequence, it has been widely used in the diagnosis of cerebrovascular diseases, brain tumours, and other neurological disorders. Dynamic susceptibility contrast (DSC) is a commonly used brain perfusion technique in clinical practice, referred to as the gold standard of magnetic resonance perfusion imaging.^5^ Experimental records involving 19 patients and 57 regions of interest, encompassing various types of tumours, were analysed to compare the CBF values measured by ASL and relative CBF values measured by DSC, and the conclusion was drawn that ASL and DSC have consistency in evaluating the CBF of brain tumours.^6^ ASL is less affected by gas and bone and does not require contrast agents, ensuring its safety and non-invasiveness compared to other techniques.

Meningiomas and cavernous sinus venous malformation commonly exhibit isointense or hypointense signals on T1-weighted imaging, while they display hyperintense signals on T2-weighted imaging.^7^ Cavernous sinus venous malformation is characterized by progressive filling enhancement.^8^ In this case, the tumour showed a slightly hypointensity on T1-weighted imaging and hyperintensity on T2-weighted imaging, no blood flow void signals were seen in the tumour, and the enhancement scan showed progressive filling enhancement, with no obvious enhancement of the surrounding meninges. Based solely on the findings from conventional MRI examination, there is a high likelihood of a cavernous sinus venous malformation. However, it is important to note that the complexity of the cavernous sinus area, as well as potential interference from gas and bone artefacts, can lead to variations in tumour appearance on routine MRI sequences. Therefore, based on the above imaging findings, it is not possible to rule out the possibility of a meningioma. Meningiomas are composed of meningothelial cells and stroma and are highly vascularized tumours in the intracranial region, often showing high perfusion,^9^ whereas cavernous sinus venous malformation are composed of enlarged capillary clusters, lacking true tumour-feeding arteries and mature drainage veins, and thus showing low perfusion.^7^^,^^10^

We conducted a comprehensive literature review and analysed 46 patients with parasellar cavernous sinus venous malformation and 45 of them are low perfusion. And all 29 cases of parasellar meningiomas exhibited high perfusion. In this case, ASL imaging revealed noticeably higher perfusion in the region of the right cavernous sinus compared to the surrounding brain cortex. Notably, this finding contradicts the majority of existing literature reports on this subject.

Cavernous sinus venous malformation may be associated with various vascular malformations, including developmental venous anomaly, capillary dilation, arteriovenous fistulas, abnormal venous dilation, and other unclassified abnormal vascular lesions. Despite not serving as the primary blood supply arteries for the tumour, arteriovenous fistulas are known to contain a marked amount of blood. Owing to the abundant arterial blood supply present in arteriovenous fistulas, the perfusion images will exhibit a marked increase in this cavernous sinus venous malformation. The histopathological examination of this patient solely identified a cavernous sinus venous malformation, and there are a couple of possible reasons for this. Firstly, it could be due to the limited range of the tissue sectioning, where the observed sections might not have included the arteriovenous malformation vessels. Secondly, the increased and enlarged capillary clusters within cavernous sinus venous malformation are difficult to distinguish from the small arteries and veins seen in arteriovenous malformations using conventional HE staining.

Each tumour has its common and characteristic manifestations, but when the lesion is located in a specific position, it may be influenced by surrounding structures, and it may not present characteristic signs. Therefore, we need to combine multiple imaging examination methods and multiple sequences to diagnose and analyse the disease.

## Learning points

- Affected by the skull base and gas, meningiomas and extracerebral cavernous malformation in the cavernous sinus area are usually difficult to distinguish, and arterial spin labelling (ASL) plays an important role in distinguishing the tumours.
- Usually the ASL of cavernous sinus venous malformation is manifested as low perfusion, while meningiomas are manifested as high perfusion.
- The patient in this case is cavernous sinus venous malformation, which presented as high perfusion on ASL, possibly due to the presence of arteriovenous fistula malformation.
